# Lifelong challenge of calcium homeostasis in male mice lacking TRPV5 leads to changes in bone and calcium metabolism

**DOI:** 10.18632/oncotarget.8779

**Published:** 2016-04-18

**Authors:** Bram C.J. van der Eerden, W. Nadia H. Koek, Paul Roschger, M. Carola Zillikens, Jan H. Waarsing, Annemiete van der Kemp, Marijke Schreuders-Koedam, Nadja Fratzl-Zelman, Pieter J.M. Leenen, Joost G.J. Hoenderop, Klaus Klaushofer, René J.M. Bindels, Johannes P.T.M. van Leeuwen

**Affiliations:** ^1^ Department of Internal Medicine, Erasmus MC, Rotterdam, The Netherlands; ^2^ Ludwig Boltzman Institute of Osteology at Hanusch Hospital of WGKK and AUVA Trauma Centre Meidling, 1st Medical Department, Hanusch Hospital, Vienna, Austria; ^3^ Department of Orthopedics, Erasmus MC, Rotterdam, The Netherlands; ^4^ Department of Physiology, Nijmegen Centre for Molecular Life Sciences, Radboud University Nijmegen Medical Centre, The Netherlands; ^5^ Department of Immunology, Erasmus MC, Rotterdam, The Netherlands

**Keywords:** TRPV5, bone resorption, mineralization, calcium homeostasis, aging, Gerotarget

## Abstract

Trpv5 plays an important role in calcium (Ca^2+^) homeostasis, among others by mediating renal calcium reabsorption. Accordingly, *Trpv5* deficiency strongly stresses Ca^2+^ homeostasis in order to maintain stable serum Ca^2+^. We addressed the impact of lifelong challenge of calcium homeostasis on the bone phenotype of these mice.

Aging significantly increased serum 1,25(OH)_2_D_3_ and PTH levels in both genotypes but they were more elevated in *Trpv5^−/−^* mice, whereas serum Ca^2+^ was not affected by age or genotype. Age-related changes in trabecular and cortical bone mass were accelerated in *Trpv5^−/−^* mice, including reduced trabecular and cortical bone thickness as well as reduced bone mineralization. No effect of *Trpv5* deficiency on bone strength was observed. In 78-week-old mice no differences were observed between the genotypes regarding urinary deoxypyridinoline, osteoclast number, differentiation and activity as well as osteoclast precursor numbers, as assessed by flow cytometry.

In conclusion, life-long challenge of Ca^2+^ homeostasis present in *Trpv5^−/−^* mice causes accelerated bone aging and a low cortical and trabecular bone mass phenotype. The phenotype of the *Trpv5^−/−^* mice suggests that maintenance of adequate circulatory Ca^2+^ levels in patients with disturbances in Ca^2+^ homeostasis should be a priority in order to prevent bone loss at older age.

## INTRODUCTION

Maintenance of adequate Ca^2+^ levels is of crucial importance for many physiological processes in the body including neuronal excitability, muscle contraction and bone formation. Bone is the major site of Ca^2+^ storage in the body, and formation and mineralization by osteoblasts as well as osteoclastic bone resorption, contribute to the maintenance of Ca^2+^ equilibrium in the circulation. Serum Ca^2+^ is tightly regulated through the concerted interactions of kidneys, intestines and bone. Transcellular Ca^2+^ (re)absorption is an important process in maintaining Ca^2+^ balance by these tissues [1, 2].

Previously, we published on the phenotype of mice lacking the epithelial Ca^2+^ channel Trpv5 (*Trpv5^−/−^*) [3]. TRPV5 is a Ca^2+^-selective transient receptor potential channel that is expressed in renal epithelial cells and crucial for reabsorption of calcium. In *Trpv5^−/−^* mice, besides hypercalciuria, intestinal Ca^2+^ hyperabsorption takes place by upregulation of the close homolog of Trpv5, Trpv6. This process is impaired when 1,25(OH)_2_D_3_ bioactivity is disturbed as shown in double knockout mice for TRPV5 and 1α-hydroxylase (synthesizes 1,25(OH)_2_D_3_) and by treatment of *Trpv5^−/−^* mice with a vitamin D receptor antagonist, ZK191784 [4, 5].

A detailed study on bone in these mice revealed that Trpv5 has a direct role in bone [6]. *Trpv5^−/−^* mice display an aberrant bone phenotype, including reduced cortical and trabecular bone thickness. Within bone, TRPV5 appears to be expressed by osteoclasts exclusively at the site where bone resorption takes place. Despite enhanced osteoclastogenesis, both *in vivo* and *in vitro*, bone resorption is seriously disturbed in mice lacking *Trpv5*, indicating that TRPV5 is required for proper osteoclast function.

It is known that increasing age is accompanied by changes in Ca^2+^ homeostasis, including reduced Ca^2+^ absorption from the diet, reduced vitamin D availability, which predisposes older persons to disorders related to Ca^2+^ homeostasis, particularly secondary hyperparathyroidism and osteoporosis [7, 8]. In addition, the capacity of 1,25(OH)_2_D_3_ to stimulate intestinal Ca^2+^ absorption declines with age, whereas circulating levels of PTH rise with age in rats and humans [9, 10]. Moreover, age-related increase in PTH levels may play an important role in changes in bone remodeling. Bone loss occurs universally with aging, leading to a reduction in bone mass and strength eventually leading to bone fragility and increased risk of osteoporotic fractures in the elderly [7, 11].

We previously demonstrated that compared to wildtype (*Trpv5^+/+^*) mice, both renal Ca^2+^ reabsorption and intestinal Ca^2+^ absorption were reduced during aging in *Trpv5^−/−^* mice, of which the latter was associated with *Trpv6* expression [12]. Moreover, elevated vitamin D receptor protein levels observed in the intestine in older mice are indicative for vitamin D resistance.

In this study we aimed to investigate the bone phenotype of aging *Trpv5^+/+^* and Trpv5^−/−^ mice by detailed analyses of serum and urine parameters related to calcium homeostasis and bone resorption, bone microarchitecture, mineralization and strength *in vivo.* Moreover, bone marrow cultures from long bones were performed to assess osteoblast and osteoclast differentiation in 78-week-old *Trpv5^+/+^* and *Trpv5^−/−^* mice. Finally, the impact of aging on calcium homeostasis and bone-related gene expression was examined in femurs and bone marrow cultures from both genotypes.

## RESULTS

### Serum 1,25(OH)_2_D_3_ and PTH are age-dependently elevated in Trpv5^−/−^ mice

**Figure 1:**
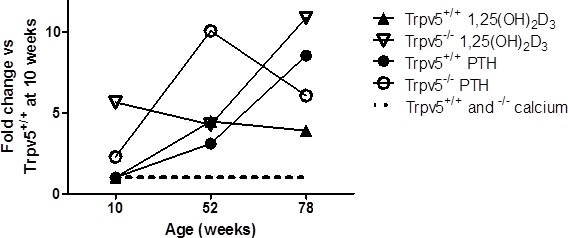
Trpv5 deficiency leads to elevated serum 1,25(OH)_2_D_3_ and PTH levels Depicted is an illustration of temporal changes in serum levels of hormones involved in calcium homeostasis. Based on the measured serum calcium levels in the 10 and 52-week old animals and calcium measured of 78-week-old male mice in this and a previous study, we have depicted calcium to be constant irrespective of age and genotype (black dotted line, not representing an actual value). Both 1,25(OH)_2_D_3_ (triangle symbols) and PTH (round symbols) levels are elevated in 10-week-old *Trpv5^−/−^* (open symbols) compared to *Trpv5^+/+^* mice (closed symbols). In fact, the serum levels for both hormones in 10-week-old TRPV5 deficient mice are similar to those in 52-week-old *Trpv5^+/+^* mice. The data presented are not longitudinal and hence should not be presented as a line graph but they serve solely as an illustration for temporal serum changes in mice lacking Trpv5. We emphasized this by disconnecting the lines from the actual measurements.

No difference in serum Ca^2+^ levels were observed between *Trpv5^+/+^* and *Trpv5^−/−^* mice at all three ages (Table 1). Serum Ca^2+^ in the oldest age group (78 weeks) was measured with a different calcium assay, which hampers direct comparison between the younger 2 age cohorts with the 78-week-old mice. However, using the same assay, previous measurements in an aging cohort of wild type mice up to 2 years of age yielded similar Ca^2+^ levels compared to the 78-week-old mice in this study (Supplementary Table 2). In *Trpv5^+/+^* mice, 1,25(OH)_2_D_3_ (pmol/l) and PTH (pg/ml) levels increased significantly with aging (Figure 1 and Table 1). In *Trpv5^−/−^* mice a similar age-related increase in serum 1,25(OH)_2_D_3_ was observed but serum 1,25(OH)_2_D_3_ levels were significantly higher in *Trpv5^−/−^* compared to their *Trpv5^+/+^* littermates at 10 and 78 weeks of age. PTH level in *Trpv5^−/−^* mice increased with age but was only significantly higher at 52 weeks age compared to *Trpv5^+/+^* mice. In 10-week-old *Trpv5^−/−^* mice, both PTH and 1,25(OH)_2_D_3_ were at a level that is not reached before 52 weeks of age in *Trpv5^+/+^* littermates (Figure 1 and Table 1).

**Table 1:** Serum measurements in *Trpv5^+/+^* and *Trpv5^−/−^* mice during aging

	10 weeks		52 weeks		78 weeks	
	*Trpv5^+/+^* *mean ± sem* *n=5*	*Trpv5^−/−^* *mean ± sem* *n=5*	*Trpv5^+/+^* *mean ± sem* *n=5*	*Trpv5^−/−^* *mean ± sem* *n=3*	*Trpv5^+/+^* *mean ± sem* *n=4*	*Trpv5^−/−^* *mean ± sem* *n=5*
***Serum***						
Calcium (mmol/l)* ^#^	2.76 ± 0.02	2.84 ± 0.02	2.71 ± 0.04	2.79 ± 0.04	2.02 ± 0.27	1.70 ± 0.29
1,25(OH)_2_D_3_ (pmol/l)	121 ± 24	686 ± 77^b^	542 ± 41	518 ± 108	474 ± 50^c^	1321 ± 133^b,d^
Parathyroid hormone (pg/ml)^#^	9.9 ± 1.9	22.6 ± 6.0	30.7 ± 6.2	100 ± 20.6^a^	84.8 ± 21.9^c^	60.2 ± 18.1

a p<0.05 vs *Trpv5^+/+^* mice of same age

b p<0.001 vs *Trpv5^+/+^* mice of same age.

c P<0.05 for age trend in *Trpv5^+/+^* mice.

d p<0.05 for age trend in *Trpv5^−/−^* mice.

* Levels at 78 weeks of age were measured by different assay compared to 10 and 52 weeks

# Published previously by Abel et al.[1]

1 van Abel, M., et al., *Age-dependent alterations in Ca2+ homeostasis: role of TRPV5 and TRPV6*. Am J Physiol Renal Physiol, 2006. **291**(6): p. F1177-83.

### Trabecular and cortical bone mass are reduced in TRPV5^−/−^ mice

**Figure 2:**
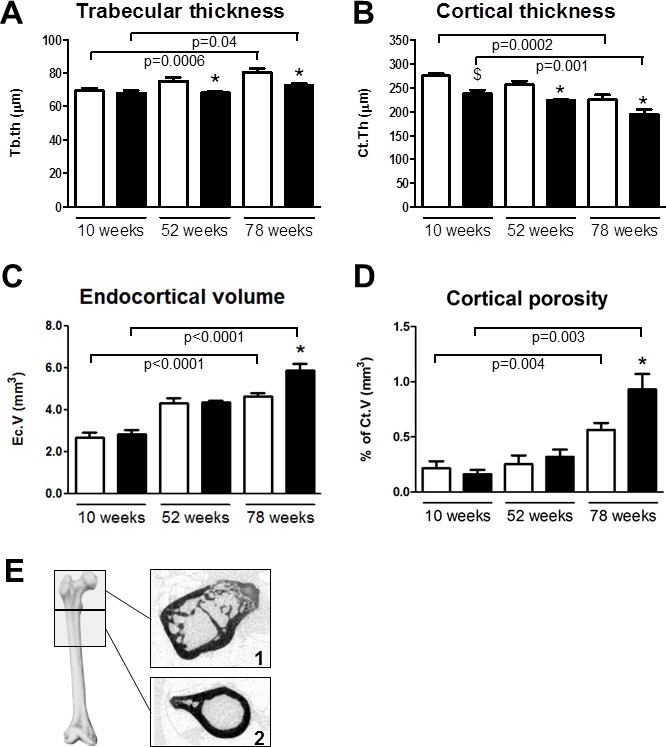
Trpv5 deficiency leads to reduced bone thickness Bone microarchitectural parameters from male *Trpv5^+/+^* (white bars) and *Trpv5^−/−^* (black bars) mice at 10, 52 and 78 weeks of age were determined by μCT analysis (*n* = 5-11). **A.** trabecular thickness in the femoral head, **B.** cortical thickness, **C.** endocortical volume and **D.** cortical porosity in the subtrochanteric area. **E.** scan regions for the calculations on trabecular (1) and cortical bone (2). Data are presented as means ± SEM. * *p* < 0.05 *versus Trpv5^+/+^*. ^$^*p* < 0.001 *vs Trpv5^+/+^*. Significant aging effects for a genotype are indicated by horizontal lines.

In *Trpv5^+/+^* mice, trabecular bone thickness (Figure 2A), trabecular spacing and structure model index (SMI) were increased in older mice, whereas trabecular number and connectivity density demonstrated an age-related decline (Supplementary Table 3). *Trpv5^−/−^* mice showed similar age-related changes as *Trpv5^+/+^* mice in the trabecular compartment although their trabecular bone thickness increased less pronounced during aging resulting in significantly lower bone mass in 52- and 78-week-old mice compared to their *Trpv5^+/+^* littermates (Figure 2A).

Within the subtrochanteric bone compartment, an age-related decrease in cortical thickness (Figure 2B) was seen in *Trpv5^+/+^* mice, while a stable cortical bone volume was maintained throughout life. Consistent with these findings, we found the endocortical volume (i.e. marrow cavity volume) to be increased with aging (Figure 2C-2D). TRPV5 deficiency led to similar age-related changes, but cortical thickness (Figure 2B) and cortical volume (Supplementary Table 3) were reduced at all ages compared to the *Trpv5^+/+^* mice. At 78 weeks of age, both endocortical volume and cortical porosity were significantly greater in the *Trpv5^−/−^* mice compared to their non-deficient littermates (Figure 2C and 2D, respectively). Other cortical parameters, such as moment of inertia and perimeter also increased during aging but they were not different between the genotypes (Supplementary Table 3).

### Mineralization of the trabecular structure is reduced in Trpv5^−/−^ mice

**Figure 3:**
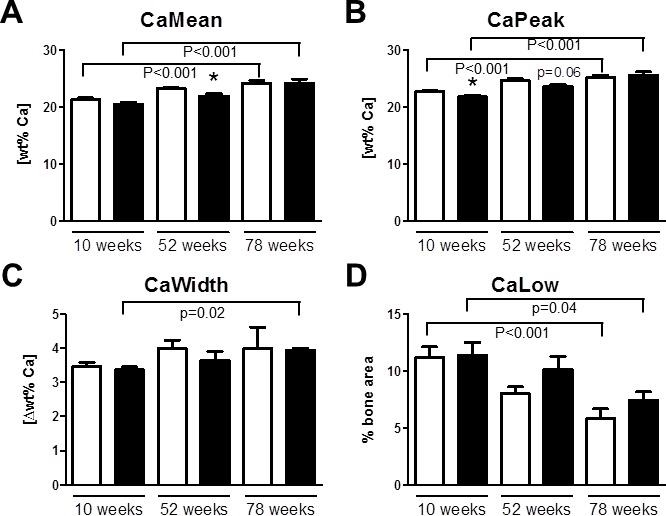
Bone mineralization is affected in *Trpv5^−/−^* mice Using quantitative backscattered electron imaging, the tibial BMDD was assessed from male *Trpv5^+/+^* (white bars) and *Trpv5*^−/−^ (black bars) mice at 10, 52 and 78 weeks of age (*n* = 3-6). Parameters were measured in the metaphyseal spongious bone compartment with respect to **A.** CaPeak, **B.** CaMean, **C.** CaWidth (all expressed as units of [wt%Ca]) and **D.** CaLow (expressed as percentage bone area). Values are presented as mean ± SEM. * *p* < 0.05 *versus Trpv5^+/+^*. ^#^*p* < 0.01 *vs Trpv5^+/+^*. ^$^*p* < 0.001 *vs Trpv5^+/+^*. Significant aging effects for a genotype are indicated by horizontal lines.

Tibial quantitative backscattered electron (qBEI) imaging showed in trabecular bone an age-related increase in the average and most abundant mineralization densities (CaMean and CaPeak, respectively) and an age-related reduction in the areas undergoing primary mineralization (CaLow) in wildtype mice (Figure 3A, 3B and 3D). The heterogeneity of mineralization (CaWidth) did not alter during aging in *Trpv5^+/+^* mice (Figure 3C). *Trpv5^−/−^* mice demonstrated age-related changes for all qBEI parameters measured being positively correlated with age for CaMean, CaPeak and CaWidth (Figure 3A, 3B and 3C, respectively), whereas CaLow was negatively correlated (Figure 3D). Both CaMean and CaPeak were reduced in the younger *Trpv5^−/−^* mice, being significant at 52 weeks and 10 weeks of age *versus Trpv5^+/+^* mice, respectively (Figure 3A and 3B, respectively). In the metaphyseal cortical bone, all parameters changed with age in both genotypes (Supplementary Table 4). In contrast to the trabecular data, in cortical bone no difference for any of the parameters at any age was observed between the genotypes (Supplementary Table 4). Both in *Trpv5^+/+^* and *Trpv5^−/−^* mice, cortical CaMean, CaPeak and CaWidth increased with age, whereas CaLow was negatively correlated (Supplementary Table 4). In the cortices of both genotypes osteocyte lacunae number and size were assessed. No difference was found for both parameters when corrected for cortical bone area (Supplementary Figure 1).

### Bone resorption in Trpv5^+/+^ and Trpv5^−/−^ bones during aging

**Figure 4:**
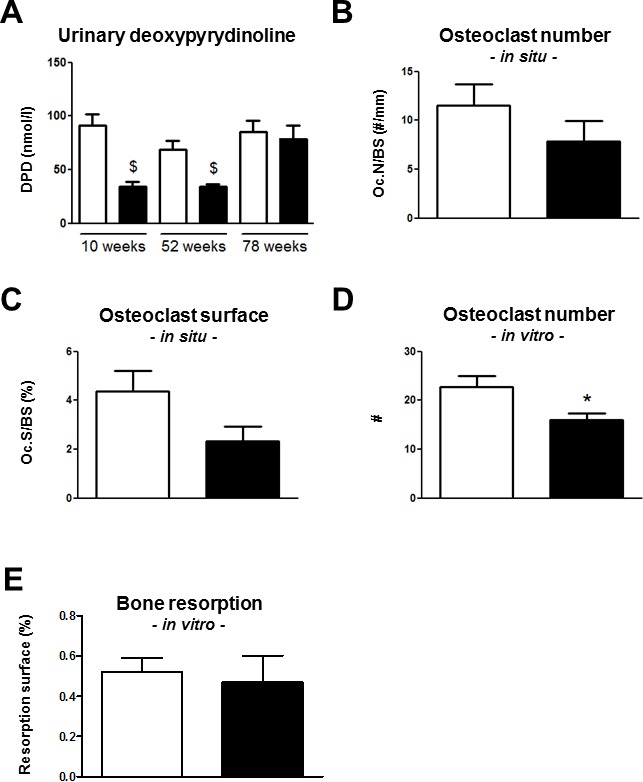
Bone resorption is reduced in *Trpv5^−/−^* mice until 52 weeks of age **A.** Urinary DPD measurement in 10, 52 and 78-week-old male *Trpv5^+/+^* (white bars) and *Trpv5*^−/−^ (black bars) mice. **B.** osteoclast number and **C.** osteoclast surface per bone surface in TRAP-stained femoral bone section of 78-week-old male *Trpv5^+/+^* and *Trpv5^−/−^* mice. **D.** osteoclast number and **E.** osteoclast surface assessed in bone marrow-derived osteoclast cultures from 78-week-old male *Trpv5^+/+^* and *Trpv5^−/−^* mice. Values are presented as mean ± SEM. * *p* < 0.05 *versus Trpv5^+/+^*. ^$^ *p* < 0.001 *vs Trpv5^+/+^*.

Bone resorption as assessed by urinary DPD was similar between the *Trpv5^+/+^* mice age groups (Figure 4A). In contrast, *Trpv5^−/−^* mice demonstrated an age-related increase in DPD levels. At 10 and 52 weeks of age, *Trpv5^−/−^* mice had significantly lower DPD levels compared to *Trpv5^+/+^* mice but at 78 weeks of age, these were similar in both genotypes (Figure 4A). Osteoclast numbers and surface in femoral bone sections of 78-week-old were not significantly different between genotypes (Figure 4B and 4C, respectively). In bone marrow cultures derived from 78-week-old mice, osteoclast numbers generated from *Trpv5^−/−^* precursor cells were significantly lower (Figure 4D), but this caused no difference in *in vitro* bone resorption (Figure 4E). Frequencies of bone marrow populations containing osteoclast precursors (i.e. immature blasts, myeloid blasts, and monocytes [13] as evaluated by flow cytometry were not different between *Trpv5^+/+^* and *Trpv5^−/−^* mice (Supplementary Table 5). The only different cell population in the bone marrow pool was the lymphoid precursor cells, which were significantly lower in the *Trpv5^−/−^* mice at 78 weeks.

### Trpv5 deficiency does not affect bone strength

Three-point-bending tests were performed on a subset of femurs from male 78-week-old *Trpv5^+/+^* and *Trpv5^−/−^* mice. There were no differences in maximum load, stiffness or energy of the femurs between the genotypes (Supplementary Figures 2A-2C). Moreover, Young's modulus, a measure for elasticity of the bone, was unaffected (Supplementary Figure 2D).

### Old Trpv5^−/−^ and Trpv5^+/+^ mice show differences in femoral gene expression patterns

Next, we generated femoral bone gene expression profiles for both genotypes at 10, 52 and 78 weeks of age by focusing at genes involved in calcium transport and homeostasis, osteoclast function, bone metabolism and phosphate homeostasis.

#### Calcium transport and homeostasis

**Figure 5:**
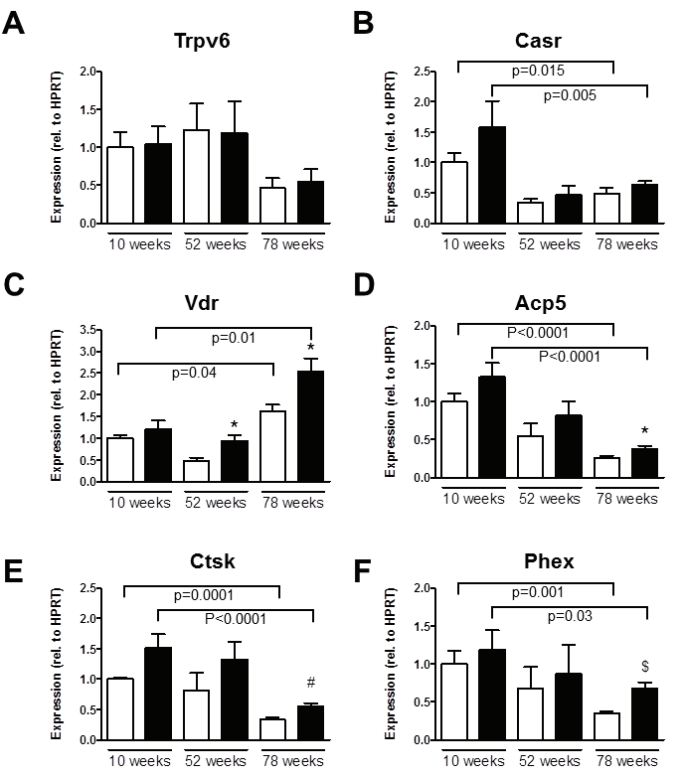
Aging *Trpv5*^−/−^ mice demonstrate high *Vdr* expression Total femoral RNA was isolated from male *Trpv5^+/+^* (white bars) and *Trpv5*^−/−^ (black bars) mice at 10, 52 and 78 weeks of age (*n* = 3-12). **A.** *Trpv6*, **B.** *Casr*, **C.** *Vdr*, **D.** *Acp5*, **E.** *Ctsk* and **F.** *Phex* mRNA. Gene expression was corrected for the housekeeping gene *Hprt* and expression in *Trpv5^+/+^* at 10 weeks was set to 1. Normalized for the housekeeping gene Values are presented as mean ± SEM. *p < 0.05 *versus Trpv5^+/+^*. ^#^ *p* < 0.01 *vs Trpv5^+/+^*.^$^ *p* < 0.001 *vs Trpv5^+/+^*. Significant aging effects for a genotype are indicated by horizontal lines.

Trpv5 mRNA was similarly expressed in femurs of 10-, 52- and 78-week-old *Trpv5^+/+^* mice (Supplementary Table 6). *Trpv6* mRNA expression in femurs of *Trpv5^+/+^* mice was lower in the 78-week-old animals compared to younger age groups, though not significant (Figure 5A). Calcium sensing receptor (*Casr)* and vitamin D receptor (*Vdr*) mRNA expression were negatively and positively correlated with age in *Trpv5^+/+^* mice, respectively (Figure 5B and 5C, respectively). Other genes involved in transcellular Ca^2+^ transport, i.e. calbindin-D_9K_ (*S100g*), sodium/calcium exchanger 1 (*Ncx1*) and plasma membrane calcium ATPase 1 (*Atp2b1*) did not change during aging of *Trpv5^+/+^* mice (Supplementary Table 6). Comparing *Trpv5^−/−^* and *Trpv5^+/+^* mice, no significant differences were observed for any of the genes related to calcium homeostasis at any time point. Besides reduced *Vdr* expression at 52 and 78 weeks of age (Figure 5C) and increased *Ncx1* expression at 78 weeks of age (Supplementary Table 6).

#### Osteoclast function

The mRNA expression of the osteoclast marker genes for tartrate-resistant acid phosphatase (*Acp5*), cathepsin K (*Ctsk*) and the calcitonin receptor (*Ctr*) was negatively correlated with age in both genotypes (Figure 5D-5E and Supplementary Table 6). All osteoclast marker genes assessed were slightly but consistently elevated in the *Trpv5^−/−^* compared to *Trpv5^+/+^* mice (Figure 5D-5E and Supplementary Table 6), but only reached significance for *Acp5* and *Ctsk*.

#### Bone metabolism and phosphate homeostasis

We also assessed several genes associated with bone metabolism and phosphate homeostasis. Among them, phosphate regulating endopeptidase homolog, X-linked (*Phex*), osteopontin (*Spp1*) and sclerostin (*Sost*)) showed reduced mRNA expression with aging in *Trpv5^+/+^* mice (Figure 5F and Supplementary Table 6). Ankylosis, progressive homolog (Ank), Fibroblast growth factor 23 (Fgf23) and Klotho (Kl) were not modulated in an age-related manner in these mice (Supplementary Table 6). As for the *Trpv5^+/+^* mice, *Phex*, *Spp1* and *Sost* expression showed a negative correlation with age in *Trpv5^−/−^* mice (Figure 5F and Supplementary Table 5). Only *Phex* expression at 78 weeks of age was significantly different in *Trpv5^−/−^* mice compared to *Trpv5^+/+^* mice (Figure 5F).

Overall, the *Trpv5^−/−^* mice showed similar age-related trends as did the *Trpv5^+/+^* mice but for some genes significantly higher expression of bone-related genes was observed at 78 weeks of age compared to the *Trpv5^+/+^* mice. In bone marrow-derived osteoblasts and osteoclasts, all marker genes studied were expressed at lower levels in the *Trpv5^−/−^* mice, but none of these differences reached significance (Supplementary Table 7).

## DISCUSSION

*Trpv5* deficiency strongly stresses the Ca^2+^ homeostasis in order to maintain stable plasma Ca^2+^ levels [3, 6]. Here, we demonstrate that mice lacking Trpv5 are able to maintain similar serum Ca^2+^ levels as their *Trpv5*-expressing counterparts during aging up to at least 78 weeks of age. This is achieved by pronounced age-related increments in serum 1,25(OH)_2_D_3_ and PTH. Age-related increases of these hormones were also observed in *Trpv5^−/−^* mice but interestingly at 10 weeks of age 1,25(OH)_2_D_3_ levels were already at the level of older (52 weeks) *Trpv5^+/+^* mice, pointing to premature aging in this respect in *Trpv5^−/−^* mice. The stress on the Ca^2+^ homeostasis is paralleled by a reduced degree of bone mineralization and accelerated changes in bone microarchitecture as exemplified by reduced bone thickness.

### Normocalcemia in Trpv5^−/−^ mice is associated with elevated serum 1,25(OH)_2_D_3_ and PTH

*Trpv5^−/−^* mice predominantly suffer from renal calcium loss, which is initially compensated by increased intestinal calcium hyperabsorption through TRPV6 followed by reduced bone mass and mineralization, to retain normocalcemia [3]. This compensatory mechanism is accompanied by elevated levels of 1,25(OH)_2_D_3_ and PTH already at 10 weeks of age. Later in life, 1,25(OH)_2_D_3_ levels further increase in *Trpv5^−/−^* mice even to levels exceeding 1300 pmol/l. These extremely high 1,25(OH)_2_D_3_ levels may explain that at 78 weeks of age the PTH levels in the *Trpv5^−/−^* are lower compared to the *Trpv5^+/+^* mice. The continuous exposure of an organism to elevated PTH and 1,25(OH)_2_D_3_ levels has adverse consequences for the skeleton. Circulating levels of PTH rise with age in rats and humans [9, 10]. From human clinical studies, it has become apparent that prolonged exposure to high PTH (p.e. primary hyperparathyroidism) indirectly increases osteoclast activity and decreases age-related osteoblast replicative activity [14-17], both contributing to bone loss.

Elevated PTH levels during human aging are at least partially caused by a decline in vitamin D levels, which is associated with reduced calcium absorption. Vitamin D levels are associated with elevated bone mineral density and reduced risk of fracture [18]. In contrast to the anabolic vitamin D effects on the skeleton in humans, Lieben *et al*. showed that exposure to high 1,25(OH)_2_D_3_ levels leads to reduced bone mineralization in mice [19]. In addition, Smith and coworkers showed that chronic high vitamin D levels in mice lead to a decrease in cortical thickness and a decline in bone stiffness [20]. In the current study, we demonstrate upregulation of the *Vdr* gene at the level of the femoral bone and strongly elevated 1,25(OH)_2_D_3_ levels in the oldest *Trpv5^−/−^* age group. Although we have no data to support this, upregulation of *Vdr* expression and high serum 1,25(OH)_2_D_3_ levels would facilitate enhanced vitamin D signaling within bone. Enhanced vitamin D signaling may thus be a mechanism to limit the influx of Ca^2+^ into bone, which would contribute to retaining serum Ca^2+^ but at the expense of bone mineralization.

Another mechanism that may contribute to retaining normocalcemia is osteocytic osteolysis. Through this process, osteocytes are capable of resorbing the lacunae around them, resulting in the liberation of ions in case of a strong Ca^2+^ demand, such as lactation [21-23]. Suffering from a chronic negative Ca^2+^ balance but keeping serum levels constant, osteocytic osteolysis in *Trpv5^−/−^* mice may suffice to restore serum Ca^2+^, which in turn would cause the observed increased cortical porosity. Although minute changes in osteocyte function may have great impact due to their numbers within bone, we failed to show detectable changes in osteocytic lacunar size or number at 78 weeks of age.

### Mineralization of bone is affected in Trpv5^−/−^ mice

In concordance with a chronic Ca^2+^ insufficiency in *Trpv5^−/−^* mice are our findings on bone mineralization density. Although no effects were seen at the level of the tibial cortex, mineralization density was decreased in the trabecular bone along with non-significantly larger areas of low mineralized bone observed in the 52- and 78-week-old *Trpv5^−/−^* mice compared to *Trpv5^+/+^* mice. As we have shown previously, the enhanced intestinal Ca^2+^ uptake could not fully compensate for the renal Ca^2+^ loss in young *Trpv5^−/−^* mice, leading to reduced skeletal mineralization [6]. Later in life, the persisting stress on Ca^2+^ homeostasis may have led to the observed increased undermineralization in old *Trpv5^−/−^* mice but also to the increased endocortical osteoclast and osteocyte activity.

### Trpv5 deficiency accelerates bone loss

Aging is associated with bone loss by thinning of cortical bone and loss of the trabecular network [24]. Indeed, we found a decrease in cortical thickness with aging and a reduction of the trabecular bone volume fraction and connectivity, despite increased thickness of the remaining trabecular bone. Previous studies looking at bone microarchitecture in aging mice found similar results [25-27]. Glatt *et al* observed an age-related decline in the trabecular bone volume fraction (BV/TV) [25], something we saw until the age of 52 weeks but not thereafter, despite a significant age trend. In line with our data, Ferguson *et al*. described cortical expansion by periosteal apposition in male mice to compensate for trabecular bone loss and reduced mineralization [26]. Hamrick and coworkers showed BMD loss, periosteal expansion and endosteal resorption up to 29 months of age [27]. Interestingly, a concurrent increase in endosteal apposition was observed at this age, for which no explanation was provided but it may be to counteract excessive endosteal bone loss. Nevertheless, it may provide an anabolic time-window for interventions to improve bone quantity/quality later in life. We did not observe differences in bone resorption between 10 and 78 weeks of life as measured by urinary DPD, which corresponds with the study by Hamrick, who showed increased bone resorption only after 18 months of age [27].

*Trpv5* deficiency led to a more accelerated bone aging phenotype, including reduced trabecular and cortical bone mass as well as increased endocortical bone volume later in life compared to *Trpv5^+/+^* mice. For example, the *Trpv5^−/−^* mice have a cortical thickness at 10 weeks of age that is not reached until *Trpv5^+/+^* at 78 weeks of age.

### Osteoclast number and bone resorption seems unaffected in 78-week-old Trpv5^−/−^ mice

In contrast to our study in young mice where we found increased numbers of osteoclasts in 10-week-old *Trpv5^−/−^* mice[6], we observed no differences in osteoclast number between *Trpv5^+/+^* and *Trpv5^−/−^* mice, but both genotypes showed lower osteoclast formation at 78 weeks compared to 52 weeks. To explain the discrepancy between the previous and current study, we assessed whether exhaustion of osteoclast precursors could have taken place in the bone marrow. However, apart from a reduction in the number of lymphoid precursor cells, we did not find a reduction in monocyte number.

Despite having a bone resorption defect in early life [6] and reduced urinary DPD levels in *Trpv5^−/−^* mice at 52 weeks, the current study showed that endocortical bone resorption in *Trpv5^−/−^* mice exceeds that of *Trpv5^+/+^* mice between 52 and 78 weeks of age, resulting in an enlargement of the marrow space (endocortical volume) at the level of the subtrochanter. In line with this would be the elevated expression levels of a few osteoclast markers in femurs of 78-week-old *Trpv5^−/−^* mice. However, bone resorption was not different between the genotypes at this age, *in vivo* and *ex vivo*, as shown by DPD levels, histomorphometry and bone marrow-derived osteoclast cultures. Apparently, old *Trpv5^−/−^* mice seem to be able to regain their bone resorption capacity, independent of TRPV5. Perhaps, prolonged high levels of circulating 1,25(OH)_2_D_3_, PTH or another yet unknown mechanism following TRPV5 deficiency has a restorative effect at the level of osteoclast activity on the skeleton of these mice. This suggests that the reduced bone resorption caused by TRPV5 deficiency that we reported previously for mice at 10 weeks of age [6] and is apparent by the decreased DPD levels at 52 weeks, may not have been entirely intrinsic and is overcome at older age.

### Bone strength is not compromised in old Trpv5 ^−/−^ mice

The combination of reduced bone mass and increased endosteal resorption suggests that life-long stress on calcium homeostasis due to TRPV5 deficiency weakens the skeleton of old mice. Besides the effects on cortical bone mass, we observed increased cortical porosity during aging but especially in the 78-week-old mice lacking TRPV5. Cortical porosity has been appreciated as an age-related phenomenon in the elderly and an important determinant of bone strength but the precise mechanism behind this process remains unclear [28]. Besides these effects on the skeleton of *Trpv5^−/−^ mice*, the 3-point-bending tests yielded similar outcomes for both genotypes at 78 weeks of age. The reason for this remains obscure, but may be due to a combination of 1) changes in the composition or the organization of the extracellular matrix leading to structural changes within the skeleton that could not be assessed through 3-point bending; 2) reduced mineralization in *Trpv5^−/−^* mice that renders bone to be more flexible; 3) non-significantly increased cortical perimeter in *Trpv5^−/−^* mice between 52 and 78 weeks of age (1.5 mm^3^) compared to *Trpv5^+/+^* mice (1.2 mm^3^) that may aid to counteract loss of bone strength. Finally, mice may possess a yet unknown ‘compensation’ mechanism that in case of bone loss protects them from a fracture, something that is rarely seen in mice.

### Human Ca^2+^ homeostasis and bone metabolism

Several disease states, such as chronic kidney diseases and intestinal malabsorption syndromes (e.g. celiac disease, intestinal surgery, aging and vitamin D deficient nutrition) in humans cause continuous stress on the maintenance of adequate serum Ca^2+^ levels, and a clear relationship has been established with bone deterioration [29-32]. The phenotype of the *Trpv5^−/−^* mice suggests that maintenance of adequate circulatory Ca^2+^ in these patients should be a priority in order to prevent bone loss and increased risk fractures at older age.

In conclusion, this study demonstrates that TRPV5 is important for normal bone development and that an unfavorable Ca^2+^ balance persisting during aging leads to accelerated bone loss in *Trpv5*-deficient mice. The strongly elevated vitamin D levels in *Trpv5^−/−^* mice may aid in restoring serum Ca^2+^ but at the expense of maintaining bone mass and/or mineralization *in vivo*. The TRPV5 deficient mouse appears to be a suitable model for lifelong challenge of calcium homeostasis and its consequences for bone metabolism.

## MATERIALS AND METHODS

### Animals

Male homozygous TRPV5 null (*Trpv5^−/−^*) and *Trpv5^+/+^* mice were generated as described before [3]. and were fed standard chow and given water *ad libitum*. At the age of 10, 52 and 78 weeks, mice were sacrificed (group sizes varied between 4-12) and serum, urine and bones were collected. The animal ethics board of the Radboud University Nijmegen approved all animal experimental procedures.

### Analytical procedures

Serum Ca^2+^ was calorimetrically determined with a Ca^2+^ assay kit (Sigma) according to the manufacturer's description at 595 nm, using a Bio-Rad microplate reader (Bio-Rad) or using a home-made calcium assay as described before [33]. Serum PTH levels were measured using a mouse intact PTH ELISA kit following standard procedure (Immutopics, San Clemente, CA, USA). Serum 1,25(OH)_2_D_3_ concentrations were measured by immunoextraction followed by quantitation by ^125^I-RIA (IDS, Boldon, UK) [6]. Urinary total deoxypyridinoline (DPD) cross-links were determined, using the Metra DPD assay according to the guidelines (Quidel).

### Microcomputed tomography (μCT)

Femurs were scanned using the SkyScan 1072 microtomograph (Bruker MicroCT) with a resolution of 7 μm and subsequently analyzed as described in detail before [6]. According to guidelines recently published [34] the following settings were used: X-Ray power and tube current were 40 kV and 0.25 mA, respectively. Beam hardening (20%) was reduced using a 1 mm aluminum filter, ring-artefacts were reduced (set at 5), exposure time was 5.9 seconds and an average of three pictures was taken at each angle (0.9°) to generate final images. Using different software packages from Bruker MicroCT (NRecon, CtAn and Dataviewer), bone microarchitectural parameters were assessed in trabecular and cortical bone of all mice (*n* = 14 for both genotypes). The trabecular bone parameters trabecular tissue volume, bone volume, trabecular volume fraction (BV/TV), trabecular thickness, trabecular number, connectivity density and structure model index were determined in the femoral head (scan area 0 - 3.25 mm of proximal femur). In the subtrochanter area (scan area 3.25 - 6.3 mm from femoral head), cortical volume, cortical thickness, cortical porosity, polar moment of inertia (MOI; proxy for bone strength) and perimeter were analyzed. For image processing, trabecular bone was manually selected and cortical bone was automatically selected. We used global thresholding for segmentation, followed by applying optimized threshold levels for trabecular and cortical bone measurements.

### Bone mechanical properties (3-point bending)

Femurs were stored in phosphate-buffered saline at −20°C until further use. Before the 3-point bending test, femurs were scanned according to the settings mentioned above. The procedure was carried out as previously described in detail [35]. Briefly, femurs were placed in a custom made 3-point bending device, with the lower loading posts 10 mm apart. Mechanical testing was performed, using a Single Column Lloyd LRX System (Lloyd Instruments). Displacement (mm) and force (N) were registered. Using the same settings for filtration, segmentation and binarization as mentioned above in the μCT section, the MOI, reflecting the ability of the bone to withstand torsion, was calculated using Ct Analyzer software (Bruker MicroCT). This was determined in the μCT scan-derived cross-section that corresponded to the fracture site resulting from the bending test. From the resulting displacement to force graphs as well as the MOI values, ultimate force (N), stiffness (N/mm), energy (Nmm) and elastic modulus (MPa) were determined as described before [36].

### Quantitative backscattered electron imaging

Dissected tibiae were routinely fixed and dehydrated in ethanol, and embedded in polymethylmethacrylate. Sample blocks were trimmed using a low speed diamond saw (Isomet-R, Buehler Ltd.). Sectioned bone surfaces were sequentially ground with sand paper with increasing grid number followed by polishing with diamond grains (size down to 1 micron) on hard polishing clothes by a precision polishing device (PM5 Logitech, Glasgow, Scotland). Finally the sample surface was carbon coated by vacuum evaporation (Agar SEM Carbon Coater) for scanning electron microscopy.

Bone mineralization density distribution (BMDD) was determined in the metaphyseal spongiosa and in the midshaft cortical bone by qBEI using a digital scanning electron microscope (DSM 962, Zeiss) equipped with a four-quadrant semiconductor backscattered electron detector as extensively described before [37, 38]. The accelerating voltage of the electron beam was set to 20 kV, the probe current to 110 pA, and the working distance to 15 mm. The cancellous and cortical bone areas were imaged at 200x nominal magnification (corresponding to a pixel resolution of 1 μm/pixel. From these digital images, grey level histograms were deduced, displaying the percentage of bone area occupied by pixels of a certain gray level. The transformation of these into calcium weight percent (wt%Ca) histograms led to a bin width of 0.17 wt%Ca. A technical precision of 0.3% was achieved. The BMDD parameters like the mean (weighted mean) CaMean and most frequently occurring calcium concentration CaPeak (peak position of the BMDD) in the sample, the width of the distribution CaWidth (full width at half maximum; Δwt%Ca) reflecting the heterogeneity in matrix mineralization and CaLow, the percentage of bone below 17.68 wt%Ca (primary mineralization)[39].

### Osteocyte density measurements

qBEI Images of cortical bone (nominal magnification 200X) with 1 μm pixel resolution were used to determine osteocyte lacunae number. The mean size was evaluated from the 2D images of the lacunae as well as the percentage of the sectioned bone surface occupied by these lacunae (density). This was done, using the software package Bioquant (Version 7.20; Bioquant image analysis corporation).

### Bone histomorphometry

After excision, femurs were routinely embedded in methylmetacrylate as described before [6]. Six μm sections were subjected to tartrate-resistant acid phosphatase (TRAP) staining. Sections were deacrylated, hydrated and rinsed in 0.2 M sodium acetate / 50 mM tartaric acid for 5 minutes. Naphtol AS-MX (0.5 mg/ml) and 1.1 mg/ml Fast red TR salt (both from Sigma) were added and incubated for 120 minutes at 37°C. Counterstaining was performed with haematoxylin for 5 seconds and after air-drying, the sections were embedded in Permount (Thermo Fischer Scientific). Images were taken with a Nikon Eclipse E400 system (Nikon, Lijnden, the Netherlands) and osteoclast number (Oc.N) and surface (Oc.S) were determined per total bone surface (BS), using Bioquant software.

### Bone marrow cultures

Bone marrow cells derived from 78-week-old mice stimulated towards osteoclasts and osteoblasts were cultured as described in detail [6, 40]. After 6 days of osteoclast culture, TRAP and coomassie brilliant blue stainings were used to stain for osteoclasts and resorption pits on bone slices left behind by osteoclasts, respectively [6]. Osteoclast number and resorption surface were determined and resorption surface per osteoclast was calculated, using the freely available ImageJ software (version 1.41; http://rsbweb.nih.gov/ij/). Osteoblast at day 14 were washed with PBS and taken up in TriZol for isolation of total RNA as described below.

### Flowcytometric analysis

Frequencies of bone marrow populations containing osteoclast precursors were determined by flowcytometry, essentially as described in de Vries *et al*. [13]. Bone marrow cells from approximately 10- and 22-month-old mice were collected, washed and counted. All immunofluorescent labelings and washes took place in FACS buffer (PBS containing 1% BSA). Bone marrow cell suspensions were spun down at 1500 rpm for 5 min at 4°C) and incubated for 30 min in 25 μl per 10^6^ cells biotinylated ER-MP12, recognizing CD31 [41]. Cells were washed once and incubated in 25 μl per 10^6^ cells FACS-buffer containing FITC-conjugated ER-MP20, recognizing Ly6C [41], and streptavidine-phycoerythrin (PE; Becton Dickinson; 10 μl per 10^6^cells) for 30 min. Finally, cells were fixed in 1% vol/vol paraformaldehyde in PBS for 15 min, washed and taken up in PBS. Cells were analyzed using a BD FACS Calibur and data were processed using Diva software (Becton Dickinson).

### RNA isolation, cDNA synthesis and real-time PCR

Pulverized material from mouse femurs (including bone marrow) was resuspended in Trizol Reagent (Gibco). RNA isolation, cDNA synthesis and real-time PCR on these femur samples as well as on the samples from the bone marrow cultures were performed as described previously [42]. Primer and probe sequences and concentrations used for real-time PCR are listed in Supplementary Table 1. The gene hypoxanthine-guanine phosphoribosyl transferase (HPRT) was used as an internal control to normalize for differences in RNA extraction and degradation as well as for efficiency of the cDNA synthesis. Data were presented as relative mRNA levels calculated by the equation 2^−ΔCt^ (ΔCt (cycle threshold) = Ct of gene of interest minus Ct of housekeeping gene).

### Statistics

In all experiments values are expressed as mean ± SEM unless stated otherwise. Differences between genotypes were tested for significance by AN(C)OVA and adjusted for age. In the *in vivo* studies, regression analyses were performed to assess age-related changes. Two-way ANOVA was performed to assess for interaction between age and genotype. Values were considered significantly different at *p* < 0.05.

## SUPPLEMENTARY MATERIAL TABLES AND FIGURES


