# Supplementary material for: Lifelong challenge of calcium homeostasis in male mice lacking TRPV5 leads to changes in bone and calcium metabolism
Source: Oncotarget. 2016 Apr 18;7(18):24928–41. doi: 10.18632/oncotarget.8779 (PMC5041880; doi:10.18632/oncotarget.8779)
Supplement: Supplementary file 1 [file oncotarget-07-24928-s001.pdf]

Lifelong challenge of calcium homeostasis in male mice lacking TRPV5 leads to changes in bone and calcium metabolism

Supplementary Material

Supplementary table 1: Murine primer sequences for SYBR-green-based Q-PCR

Gene	Forward (5'-3')	Reverse (5'-3')	Quantity (pmol)
Hprt	TTATCAGACTGAAGAGCTACTGTAATGATC	TTACCAGTGTCAATTATATCTTCAACAATC	2.5
Trpv5	CGTTGGTTCTTACGGGTTGAAC	GTTTGGAGAACCACAGAGCCTCTA	2.5
Trpv6	TTCCAGCAACAAGATGGCCTCTACTCTGA	ATCCGCCGCTATGCACA	10
S100g	CCTGCAGAAATGAAGAGCATTTT	CTCCATCGCCATTCTTATCCA	10
Ncx1	TCCCTACAAAACTATTGAAGGCACA	TTTCTCATACTCCTCGTCATCGATT	10
Atp2b1	CGCCATCTTCTGCACCATT	CAGCCATTGCTCTATTGAAAGTTC	10
Ctsk	TGATGAAAATTGTGACCGTGATAA	CTCTCTCCCCAGCTGTTTTTAATTA	2.5
Clcn7	CCTGTGGTGGAGGATGTAGGA	TCTCCACAAACACCTTATGCTT	5
Acp5	AAGAACTTGCGACCATTGTTAGC	CCTGAAGATACTGCAGGTTGTGG	2.5
Tcirg1	TCAGATCCTAAGCCGAAGTTGAG	GACCAAGGCCACCTCTTCAC	5
Vdr	GATCTTGTCAGTTACAGCATCCAAAA	CGCAACATGATCACCTCAATG	2.5
Casr	CTTTCCTATCCATTTTGGAGTAGCA	GCAAAGATCATGGCTTGTAACCA	10
Ctr	GTGATGAAAATGGAGAGTGGTTTAGA	AAGAACGTACGCATTTTGCAGT	2.5
Kl	CCAAAAGCTGATAGAGGACAATGG	GTGTCCACTTGAACGTAGTTGTCAA	5
Sost	ACCTCCCCACCATCCCTATG	TGTCAGGAAGCGGGTGTAGTG	2.5
Ankh	TACAGAGGCAGTGGCCATTCT	GTTGCTTGTGTTCGCCAGTTT	5
Spp1	AAGGATGACTTTAAGCAAGAAACTCTTC	TCCTCGCTCTCTGCATGGT	2.5
Fgf23	CATCTACAGTGCCCTGATGATTACA	CTTCGAGTCATGGCTCCTGTT	5
Phex	AACCGAACCAGTGAGGCTATGT	TAGAGTCTGGTATCAATGACCTTCTTG	2.5

Supplementary table 2: **Serum calcium levels in 18-month-old Trpv5+/+ mice.**

	WT mice previous cohort
	**WT**
	mean ± sem
Serum	
Calcium (mmol/l)	2.56 ± 0.04

Supplementary table 3: µCT data of femurs from male *Trpv5*$^{+/+}$ and *Trpv5*$^{-/-}$ mice during aging

	10 weeks		52 weeks		78 weeks	
	Trpv5$^{+/+}$ *mean ± sem*	***Trpv5***$^{-/-}$ *mean ± sem*	***Trpv5***$^{+/+}$ *mean ± sem*	***Trpv5***$^{-/-}$ *mean ± sem*	***Trpv5***$^{+/+}$ *mean ± sem*	***Trpv5***$^{-/-}$ *mean ± sem*
Femoral head trabecular bone						
Trabecular thickness (µm)	69.5 ± 1.5	68.0 ± 1.6	75.2 ± 2.3	68.1 ± 0.8 [a]	80.3 ± 2.5 [d]	72.5 ± 1.5 [a,e]
Trabecular volume (mm^3)	1.5 ± 0.1	1.3 ± 0.1	0.9 ± 0.0	1.0 ± 0.1	1.2 ± 0.1	1.2 ± 0.1
Trabecular bone volume fraction (BV/TV; %)	30.6 ± 1.7	26.4 ± 1.5	16.6 ± 1.1	18.4 ± 2.5	20.6 ± 1.4	19.5 ± 2.0
Trabecular number (mm^{-1})	0.73 ± 0.02	0.75 ± 0.02	0.41 ± 0.04	0.53 ± 0.06 [a]	0.46 ± 0.05 [d]	0.50 ± 0.04 [e]
Trabecular separation (µm)	271 ± 8	285 ± 9	383 ± 16	380 ± 45	407 ± 21 [d]	391 ± 26 [e]
Connectivity density (mm^{-1})	449 ± 59	317 ± 17	51 ± 16	121 ± 43	89 ± 24 [d]	122 ± 33 [e]
Structure model index	1.6 ± 0.1	1.7 ± 0.1	2.1 ± 0.1	1.9 ± 0.1	1.9 ± 0.1 [d]	2.0 ± 0.1 [e]
Subtrochanter cortical bone						
Cortical volume (mm^3)	4.0 ± 0.2	3.4 ± 0.2 [a]	4.1 ± 0.1	3.7 ± 0.1 [a]	4.1 ± 0.2	3.6 ± 0.1 [a]
Cortical thickness (µm)	275 ± 6	238 ± 7	256 ± 8	224 ± 2	225 ± 10 [d]	195 ± 10 [e]
Endocortical volume (mm^3)	2.7 ± 0.2	2.8 ± 0.2	4.3 ± 0.2	4.4 ± 0.1	4.6 ± 0.2 [d]	5.9 ± 0.3 [e]
Cortical porosity (% of Ct.V)	0.22 ± 0.06	0.16 ± 0.04	0.25 ± 0.08	0.32 ± 0.06	0.56 ± 0.06 [d]	0.93 ± 0.14 [a,e]
Polar moment of inertia (mm^4)	0.68 ± 0.07	0.54 ± 0.06	0.90 ± 0.05	0.83 ± 0.05	1.02 ± 0.05 [d]	1.04 ± 0.05 [e]
Perimeter (mm)	5.6 ± 0.1	5.32 ± 0.15	6.4 ± 0.3	6.3 ± 0.4	7.6 ± 0.1 [d]	7.8 ± 0.2 [e]

[a] $p<0.05$ versus *Trpv5*$^{+/+}$ mice of the same age, [b] $p<0.01$ versus *Trpv5*$^{+/+}$ mice of the same age, [c] $p<0.001$ versus *Trpv5*$^{+/+}$ mice of the same age. [d] $p<0.05$ for age trend in *Trpv5*$^{+/+}$ mice. [e] $p<0.05$ for age trend in *Trpv5*$^{-/-}$ mice.

Supplementary table 4: qBEI data of cortical bone from male *Trpv5*$^{+/+}$ and *Trpv5*$^{-/-}$ mice during aging

	10 weeks		52 weeks		78 weeks	
	Trpv5$^{+/+}$ *mean ± sem*	***Trpv5***$^{-/-}$ *mean ± sem*	***Trpv5***$^{+/+}$ *mean ± sem*	***Trpv5***$^{-/-}$ *mean ± sem*	***Trpv5***$^{+/+}$ *mean ± sem*	***Trpv5***$^{-/-}$ *mean ± sem*
Diaphyseal cortex						
CaMean (wt% Ca)	22.8 ± 0.3	23.3 ± 0.4	25.6 ± 0.2	24.5 ± 0.3	26.1 ± 0.6 [a]	26.6 ± 0.2 [b]
CaPeak (wt% Ca)	23.7 ± 0.3	24.1 ± 0.4	26.5 ± 0.2	25.5 ± 0.3	27.0 ± 0.6 [a]	27.4 ± 0.3 [b]
CaWidth (Δwt% Ca)	3.12 ± 0.09	3.02 ± 0.09	2.98 ± 0.17	3.06 ± 0.12	4.39 ± 0.35 [a]	3.70 ± 0.21 [b]
CaLow (% bone area)	4.49 ± 0.45	3.76 ± 0.33	3.17 ± 0.11	3.51 ± 0.39	2.32 ± 0.42 [a]	2.38 ± 0.37 [b]

[a] p<0.05 for age trend in *Trpv5*$^{+/+}$ mice. [b] p<0.05 for age trend in *Trpv5*$^{-/-}$ mice.

Supplementary table 5: Mouse bone marrow composition analyzed by flowcytometry in *Trpv5*$^{+/+}$ and *Trpv5*$^{-/-}$ mice[1]

		10 months		22 months	
	phenotype	***Trpv5*$^{+/+}$**	***Trpv5*$^{-/-}$**	***Trpv5*$^{+/+}$**	***Trpv5*$^{-/-}$**
Cell population (%)		*mean ± sem*	*mean ± sem*	*mean ± sem*	*mean ± sem*
Early blasts	$CD31^{hi}$ $Ly\text{-}6C^{neg}$	1.5 ± 0.2	1.6 ± 0.0	1.3 ± 0.3	1.3 ± 0.1
Lymphoid cells	$CD31^{int}$ $Ly\text{-}6C^{neg}$	17.3 ± 1.9	17.4 ± 2.8	19.5 ± 1.5	10.7 ± 1.2 [a]
Erythroid precursors	$CD31^{neg}$ $Ly\text{-}6C^{neg}$	16.8 ± 1.8	18.9 ± 2.0	14.2 ± 0.9	15.3 ± 1.8
Myeloid blasts	$CD31^{pos}$ $Ly\text{-}6C^{pos}$	7.8 ± 0.2	6.5 ± 0.8	8.2 ± 0.2	8.9 ± 0.8
Neutrophils	$CD31^{neg}$ $Ly\text{-}6C^{int}$	22.6 ± 1.4	23.4 ± 1.8	24.1 ± 1.0	29.2 ± 2.4
Monocytes	$CD31^{neg}$ $Ly\text{-}6C^{hi}$	5.6 ± 0.6	3.5 ± 1.0	4.6 ± 0.5	5.5 ± 0.3

[a] $p<0.05$ versus *Trpv5*$^{+/+}$ mice of the same age

1 Bone marrow populations were identified as described in de Vries *et al*. 2009 [1].

Supplementary table 6: Gene expression in femurs from *Trpv5*$^{+/+}$ and *Trpv5*$^{-/-}$ mice during aging

	10 weeks		52 weeks		78 weeks	
	Trpv5$^{+/+}$ *mean ± sem*	***Trpv5***$^{-/-}$ *mean ± sem*	***Trpv5***$^{+/+}$ *mean ± sem*	***Trpv5***$^{-/-}$ *mean ± sem*	***Trpv5***$^{+/+}$ *mean ± sem*	***Trpv5***$^{-/-}$ *mean ± sem*
Calcium homeostasis						
Trpv5	1.0 ± 0.2	-	1.0 ± 0.2	-	0.8 ± 0.1	-
Calbindin-D_{9K} (*S100g*)	1.0 ± 0.1	1.6 ± 0.2	0.7 ± 0.1	0.7 ± 0.1	1.0 ± 0.2	0.8 ± 0.2 [c]
Sodium/calcium exchanger 1 (*Ncx1*)	1.0 ± 0.2	1.1 ± 0.1	0.8 ± 0.1	0.8 ± 0.2	1.0 ± 0.1	1.4 ± 0.1 [a]
Plasma membrane calcium ATPase 1 (*ATP2b1*)	1.0 ± 0.1	0.8 ± 0.1	1.1 ± 0.2	1.4 ± 0.3	0.9 ± 0.1	1.3 ± 0.2
Osteoclast markers						
Calcitonin receptor (*Ctr*)	1.0 ± 0.2	1.1 ± 0.2	0.8 ± 0.3	1.3 ± 0.3	0.4 ± 0.1 [b]	0.5 ± 0.1 [c]
Chloride channel 7 (*Clcn7*)	1.0 ± 0.1	1.3 ± 0.2	0.7 ± 0.1	1.0 ± 0.1	1.2 ± 0.1	1.2 ± 0.1
H^+-ATPase (*Tcirg1*)	1.0 ± 0.1	1.2 ± 0.2	0.7 ± 0.1	1.0 ± 0.1	1.2 ± 0.1	1.4 ± 0.1
Aging of bone						
Osteopontin (*Spp1*)	1.0 ± 0.0	1.1 ± 0.0	0.8 ± 0.2	0.9 ± 0.2	0.5 ± 0.1 [b]	0.7 ± 0.1 [c]
Sclerostin (*Sost*)	1.0 ± 0.2	1.6 ± 0.4	0.5 ± 0.1	0.5 ± 0.1	0.6 ± 0.0 [b]	0.7 ± 0.1 [c]
Ankylosis, progressive homolog (*Ank*)	1.0 ± 0.2	1.0 ± 0.2	0.6 ± 0.1	0.6 ± 0.1	0.7 ± 0.1	0.8 ± 0.0
Fibroblast growth factor 23 (*Fgf23*)	1.0 ± 0.3	1.5 ± 0.4	3.1 ± 0.7	1.4 ± 0.3	1.7 ± 0.3	1.8 ± 0.5
Klotho (*Kl)*	1.0 ± 0.3	0.7 ± 0.1	1.4 ± 0.3	0.8 ± 0.3	1.1 ± 0.3	1.0 ± 0.3

Data were normalized to *Trpv5*$^{+/+}$ mice at 10 weeks of age (set to 1.0). [a] $p<0.05$ vs *Trpv5*$^{+/+}$ mice of same age. [b] $p<0.05$ for age trend in *Trpv5*$^{+/+}$ mice. [c] $p<0.05$ for age trend in *Trpv5*$^{-/-}$ mice.

Supplementary table 7: Gene expression in *Trpv5*$^{+/+}$ and *Trpv5*$^{-/-}$ bone marrow-derived osteoblasts and osteoclasts

	**78 weeks**	
	Trpv5$^{+/+}$ *mean ± sem*	***Trpv5***$^{-/-}$ *mean ± sem*
Osteoblast genes		
Collagen 1a1 (*Col1a1*)	108.2 ± 43.9	61.1 ± 9.7
Osteocalcin (*Bglap*)	0.58 ± 0.31	0.22 ± 0.05
Runt-related transcription factor 2 (*Runx2*)	0.48 ± 0.19	0.28 ± 0.02
Osteoclast genes		
Acid phosphatase 5, tartrate resistant *(Acp5*)	39.1 ± 10.7	34.1 ± 7.2
Chloride channel, voltage-sensitive 7 (*Clcn7*)	0.32 ± 0.08	0.28 ± 0.04
T-Cell, Immune Regulator 1, ATPase, H+ Transporting, Lysosomal V0 Subunit A3 (*Tcirg1*)	1.53 ± 0.39	1.22 ± 0.29

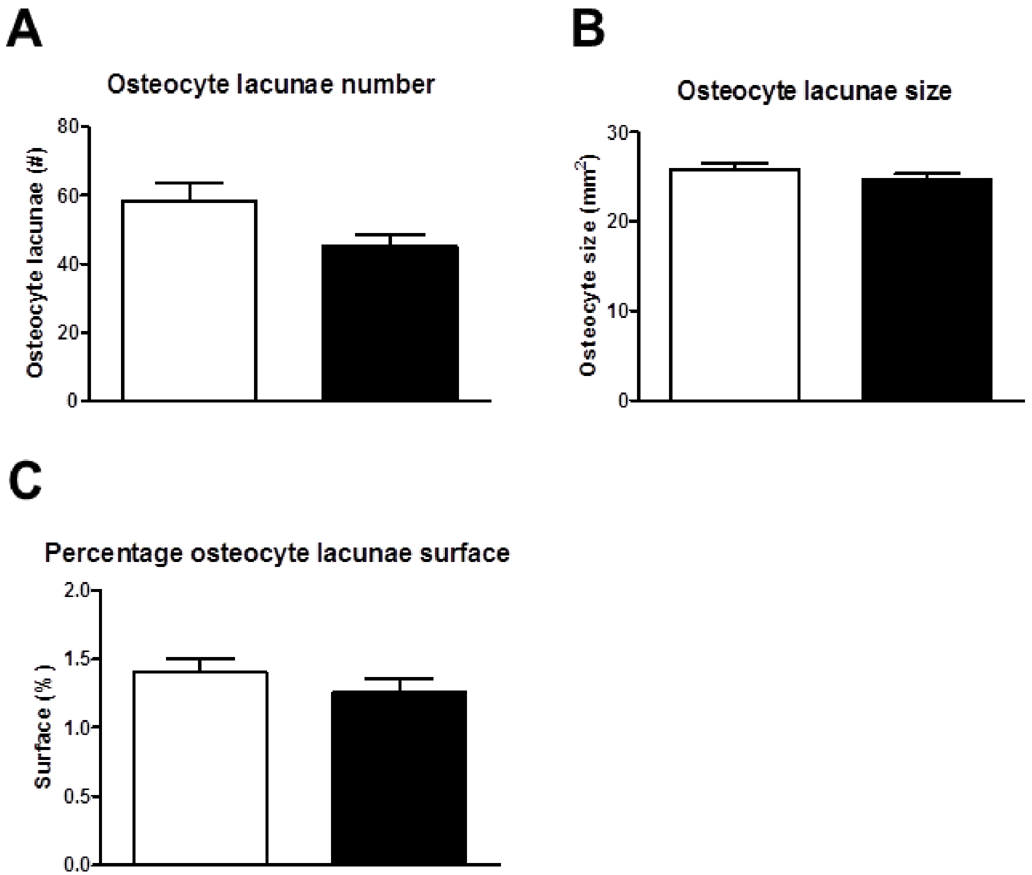

Supplementary figure 1: Osteocyte lacunae are not affected in old ***Trpv5***$^{-/-}$ **m**ice

Femoral bone sections of male *Trpv5*$^{+/+}$ (white bars) and *Trpv5*$^{-/-}$ (black bars) mice were used to determine A) osteocyte lacunae number, B) osteocyte lacunae size and C) percentage osteocyte lacunae surface/bone surface. Values are presented as mean ± SEM.

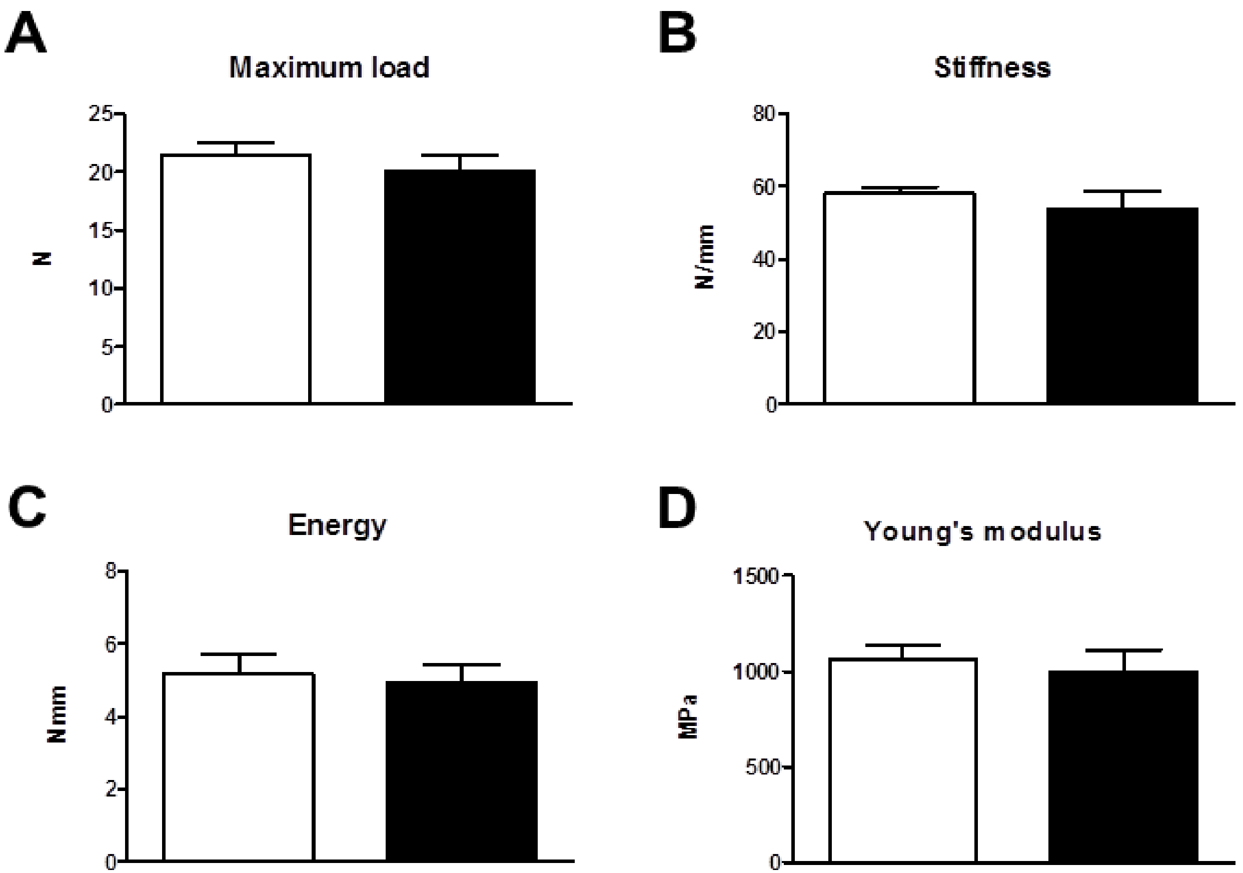

Supplementary figure 2: Bone strength is not affected in **old *Trpv5*** $^{-/-}$ mice

Femurs of male *Trpv5* $^{+/+}$ (white bars) and *Trpv5* $^{-/-}$ (black bars) mice were subjected to 3-point-bending (n=6-9). A) Maximum load, B) Stiffness, C) Energy and D) Young's modulus were measured. Values are presented as mean ± SEM.

References

1. de Vries TJ, Schoenmaker T, Hooibrink B, Leenen PJ and Everts V. Myeloid blasts are the mouse bone marrow cells prone to differentiate into osteoclasts. J Leukoc Biol. 2009; 85(6):919-927.
